# In vivo medical imaging for assessing geroprotective interventions in humans

**DOI:** 10.1007/s11357-025-01514-y

**Published:** 2025-02-06

**Authors:** Jonas E. Svensson, Martin Schain, Pontus Plavén-Sigray

**Affiliations:** 1 Department of Clinical Neuroscience, Karolinska Institutet, Stockholm, Sweden; 2 Theme Inflammation and Aging, Karolinska University Hospital, Stockholm, Sweden; 3 Antaros Medical AB, Mölndal, Sweden; 4 Neurobiology Research Unit, Copenhagen University Hospital, Rigshospitalet, Copenhagen, Denmark

**Keywords:** Biomarker, Geroprotection, Drug development, Age-related disease, Imaging, PET, CT, MRI, OCT

## Abstract

There is a growing interest in developing drugs with a general geroprotective effect, aimed at slowing down aging. Several compounds have been shown to increase the lifespan and reduce the incidence of age-related diseases in model organisms. Translating these results is challenging, due to the long lifespan of humans. To address this, we propose using a battery of medical imaging protocols that allow for assessments of age-related processes known to precede disease onset. These protocols, based on magnetic resonance imaging, positron emission-, computed-, and optical coherence tomography, are already in use in drug development and are available at most modern hospitals. Here, we outline how an informed use of these techniques allows for detecting changes in the accumulation of age-related pathologies in a diverse set of physiological systems. This in vivo imaging battery enables efficient screening of candidate geroprotective compounds in early phase clinical trials, within reasonable trial durations.

## Introduction

The past half century has witnessed a gradual increase in aging research and the development of anti-aging interventions. Since the 1930s, it has been known that the lifespan in mammals can be extended using caloric or dietary restriction [1]. In 2009, it was demonstrated that a pharmacological intervention with the drug rapamycin elicited increased lifespan in rodents [2]. This sparked an interest in the development of new pharmacological compounds for geroprotection, so-called candidate gerotherapeutics or geroprotective interventions [3] (CGPIs; see Table 1). Today, a large number of pharmaceutical and biotech companies are pursuing this avenue, and a multitude of CGPIs have been proposed and entered testing in animals [5, 6].

**Table 1:** Definition of terms used in the review

Term	Definition
Aging	The process of accumulation of the consequences of life over time, such as molecular and cellular damage, that leads to functional decline, morbidity and ultimately mortality. *
Biological age	This conceptually defines an individual’s age based on the level of age-dependent biological changes, such as accumulation of molecular and cellular damage over time. *
Chronological age	An individual’s age defined by the time elapsed since birth. *
Geroprotective intervention (GPI)	A drug or intervention that inhibits or postpones age-related functional decline, morbidity and mortality. *
Surrogate endpoint	A measure that is known or likely to predict clinical benefit and could be used to support regulatory approval of a drug or biological product
Biological plausibility	The concept of whether a likely causal relationship exists between exposure to a pathological process and a health outcome based on existing knowledge

*Definition adopted from Moqri et al. [4]

Despite promising preclinical results, CGPIs have rarely been tested in humans. One major reason for this lack of translational research is the lengthy trial duration needed to evaluate their impact on human longevity. A study assessing geroprotective effects, and using mortality as primary endpoint, would have to run over several decades in order to determine whether the treatment is effective. The vast resources required to complete such a trial currently prevent CGPIs from entering phase II or III testing in humans [7].

The only large-scale clinical trial involving CGPIs currently being planned is the TAME (Targeting Aging With Metformin) study, where the geroprotective effects of the diabetes medication metformin will be evaluated [8]. Rather than mortality, the researchers will use the onset of several age-related disorders (i.e., cancer, type 2 diabetes, cardiovascular disease, and dementia) as primary endpoints. Based on the *geroscience hypothesis* [9]*,* the rationale is that the onset of all age-related diseases will be delayed if the CGPI works. A successful GPI will target one, or a few, fundamental biological process(es) that drive aging, and thereby inhibit or reverse downstream effects that lead to age-related morbidity and mortality. In conditions where risk factors aside from aging play an important role (such as diet in type 2 diabetes or smoking in cardiovascular disease), the hypothesis states that the biological aging process will accelerate the accumulation of pathology beyond that which is caused by lifestyle factors alone. Thus, an effective GPI should delay the onset of such diseases as well.

Although the strategy employed in TAME involves a substantial reduction in trial duration compared to a study using mortality as primary endpoint, it is not ideal for the purpose of screening for CGPIs in humans. A more efficient screening process would be valuable if it could, in an even shorter duration and using a smaller sample size, provide information on whether the candidate treatment should be advanced to subsequent phases or if development should be discontinued.

A single definite biomarker for an individual’s biological age would be ideal in this context. Several fluid-based candidate biomarkers have been proposed for this purpose, including studying the accumulation of methyl groups on the genome, so-called epigenetic clocks. These clocks show high precision in predicting chronological age [10, 11]. It is less established, however, how well they predict age-related morbidity and mortality [12]. In the setting of clinical trials, an important criterion for a biomarker used as a suitable endpoint is to exhibit “biological plausibility”, hence reflecting a cause-and-effect relationship to a particular disease [13]. This is challenging for many fluid-based biomarkers, including epigenetic clocks, given the difficulties in interpreting the mechanisms behind their provided outcomes.

Here, we propose an alternative strategy to screen CGPIs for efficacy. In line with the guiding principle of the TAME trial, it is assumed that a successful GPI will delay the onset of age-related disorders. In contrast to the TAME trial, however, we propose to assess the underlying pathological processes that precede the actual onset of diseases. More specifically, in most age-related disorders, the disease onset follows several years of accumulation of pathology. Thus, if a CGPI can delay the onset of an age-related disease, it should also attenuate or revert the preceding underlying pathological process. To this end, we propose using in vivo medical imaging (see Box 1) to assess age-related pathology in several tissues in parallel in early-phase evaluations of CGPIs. Such an imaging battery would allow for shorter, cheaper, and more efficient trials compared to the strategy employed in the TAME trial. Similar to the TAME design, however, it puts the emphasis on age-related pathology, in contrast to studies using aging biomarkers without a clear connection to morbidity or mortality.

**Box 1** Explanation of the proposed in vivo imaging modalities

**Table:** 

**PET** Positron Emission Tomography (PET) relies on intravenous administration of a radiolabeled molecule (radioligand) to a patient. The PET system measures the radiation emitted by the label, enabling visualization and quantification of the radioligand’s distribution in tissue. The most common radioligand, [^18^F]FDG, is a radiolabeled glucose analogue, and its use informs on glucose metabolism in various tissues
**MRI** Magnetic Resonance Imaging (MRI) uses strong magnetic fields and radio waves to map hydrogen content in tissue in a patient. The MR system generally exhibits excellent soft tissue contrast, with specific pulse sequences available for estimating tissue perfusion, oxygenation, and regional blood flow
**CT** Computed tomography (CT) scanners use a rotating source emitting X-ray radiation. The patient is positioned in a circular array of detectors that captures the radiation that has propagated through the body. A detailed 3D image of the anatomy can then be created. During a quantitative CT (qCT) examination, the readout from the patient can be cross-compared to that of a density phantom to enable precise estimation of, e.g., bone mineral density
**DXA** During a Dual-Energy X-Ray Absorptiometry (DXA) scan, two X-ray beams are passed through a selected region of a patient, and unabsorbed photons are detected to create a pixel-by-pixel map of bone, fat, and lean tissues. This information is useful for measuring bone mineral density and relative fat/muscle content (i.e., body composition)
**OCT** Retinal Optical Coherence Tomography (OCT) captures the back-propagating reflections of dim red light that has been emitted into the eye. This technique enables non-invasive and high-resolution measurements of sub-surface structures, including the retinal nerve fiber layer

Medical imaging has evolved greatly over past decades, and an informed use of the various techniques that are available at most hospitals could allow for early detection of whether the CGPI attenuates or inhibits age-related pathological processes. Such a finding would give support for the treatment possessing geroprotective properties. Conversely, if the battery, in a well powered study, detects no effect on any of the assessed age-related pathological processes, this provides support for an inefficient treatment. Resources can then be re-routed to the development of other drug candidates.

In the following sections, we will introduce the concept of using medical imaging to assess CGPI efficacy in humans. To this end, we outline a set of criteria for identifying suitable age-related pathological processes and propose imaging protocols that can be used in tandem to assess the respective processes, prior to the onset of manifest disease. We finally discuss different considerations that should be taken into account when researchers design their own imaging batteries to be deployed in future trials of promising CGPIs.

## Criteria for efficient screening of CGPIs

For a medical imaging battery to be useful for efficient CGPI screening, the various pathological processes evaluated must be:

- Sufficiently diverse (i.e., the battery must tap into several distinctly different age-related processes for it to evaluate a *general* geroprotective effect).

Further, any individual pathological process assessed by the battery must be:

- Associated with chronological age (e.g., predictably increase with progressing age).
- Causally linked to one or several age-related disorders, so that:
  o The degree of pathology predicts the risk and/or the severity of the disorder(s).
  o The disorder(s) is/are unlikely to occur in the absence of the pathological process.

Furthermore, the different imaging protocols of the battery must:

- Have sufficient longitudinal reliability (i.e., be sensitive enough to detect changes in a pathological process over time intervals and sample sizes that are relevant in the context of clinical trials).
- Be measurable in human research subjects without unacceptable hazard, discomfort, labor, or expense.

## Proposed protocols of the imaging battery

**Fig. 1:**
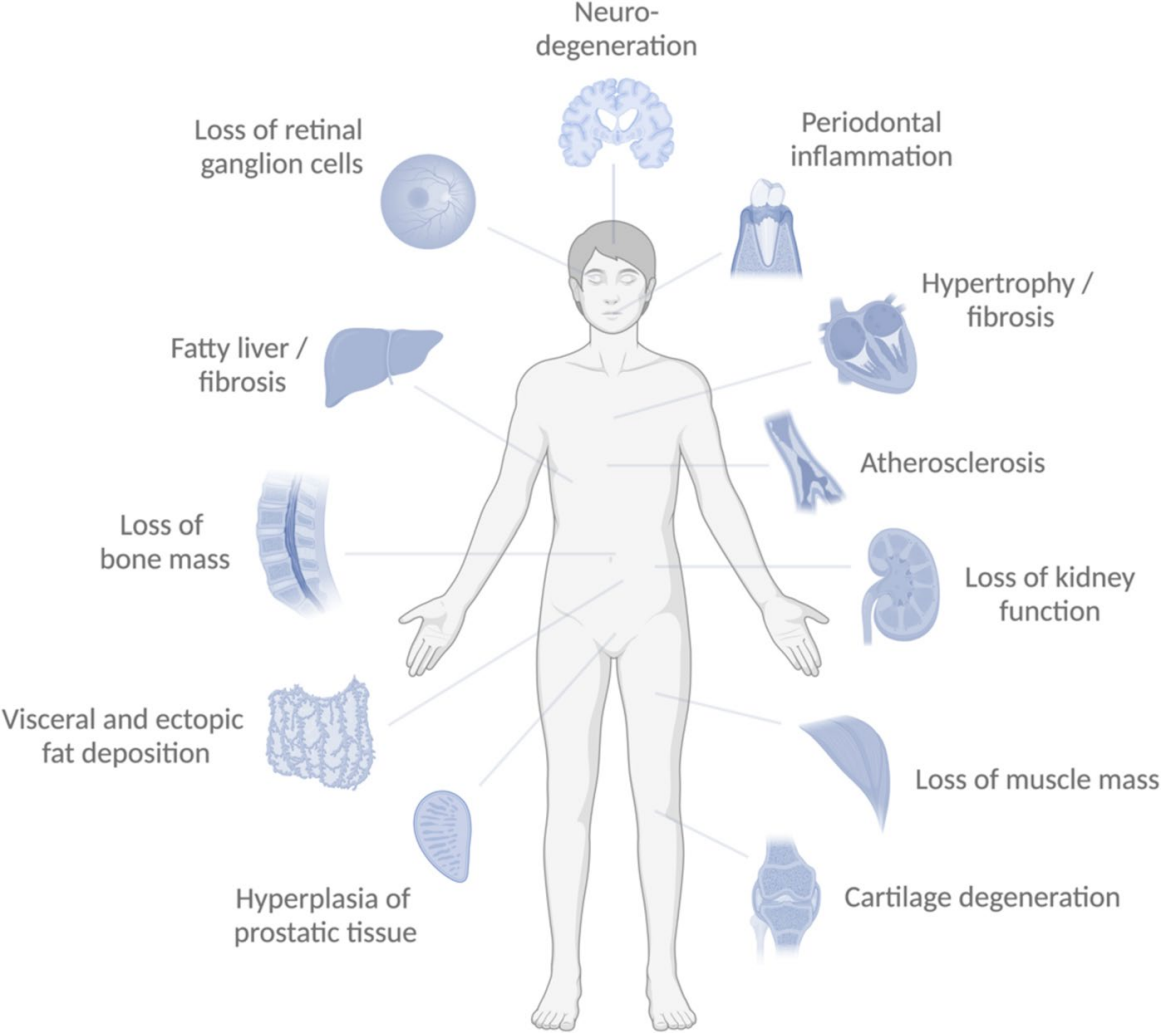
Schematic over-included tissues and organs where age-related pathological processes are known to cause to morbidity and mortality. Image-based assessments of these processes are listed in Table 2

Figure 1 depicts pathological processes that, if left unchecked, are likely to lead to age-related morbidity. Table 2 lists useful protocols for imaging these processes, of which a subset could be combined into a battery and used in a clinical trial. These protocols assess morphological or functional changes that are central to the pathological processes that underlie, and precede, age-related disorders. Treatment with an effective GPI can be expected to reduce or reverse the progression of several of these pathological processes.

**Table 2:** List of available imaging protocols that enable assessment of morphological and functional changes caused by pathological processes that are predictive of later onset of age-related disorders

Physiological system	Age-related disorder	Pathological process	Quantifiable change	Imaging protocol
Cardiovascular	Cardiac and cerebral infarction / angina pectoris / peripheral artery disease	Atherosclerosis	Vessel stiffness Plack inflammation Calcification	MRI Pulse wave velocity/ MRI PIVOT / T2* [^18^F]FDG PET CT
	Heart failure	Hypertrophy / fibrosis	Myocardial volume, diastolic function, “strain”	Cardiac MRI, echocardiography
Musculoskeletal	Osteoarthritis	Cartilage degeneration	Joint cartilage volume	3D T1W MRI
	Osteoporosis	Loss of bone mass	Bone mineral density	DXA, quantitative CT, [^18^F]NaF PET
	Sarcopenia	Loss of muscle mass	Muscle volume or density/fat infiltration	DXA, quantitative CT, MRI body composition,^1^H-MRS
Metabolism	Metabolic syndrome	Visceral and ectopic fat deposition	Body composition	DXA, quantitative CT, MRI body composition, ^1^H-MRS
	Type 2 Diabetes	Insulin resistance	Glucose metabolism	[^18^F]FDG PET
	MASH / MAFLD / hepatocellular carcinoma / cirrhosis	Fatty liver / fibrosis	Tissue stiffness, fibrotic load and formation	MRE, [^68^ Ga]FAPI PET, [^68^ Ga]PDGFRβ PET
Neurological/ophthalmological	Dementia disorders	Neurodegeneration	Gray matter volume, glucose metabolism	T1w Brain MRI/ [^18^F]FDG PET
	Macular degeneration	Drusen accumulation	Drusen concentration	Retinal OCT
	Glaucoma	Loss of retinal ganglion cells	Thickness of retinal nerve fiber layer	Retinal OCT
Dental	Periodontitis	Periodontal inflammation and loss of tooth-bearing bone	Glucose metabolism / oedema volume / bone loss	[^18^F]FDG PET, T2 STIR MRI, CT
Urological/nephrological	Benign prostatic hyperplasia	Hyperplasia of prostatic tissue	Volume of prostatic gland	3D MRI
	Chronic Kidney Disease / Kidney Failure	Loss of kidney function	Blood flow / volume / filtration fraction / diffusion / oxygenation	Multiparametric MRI

*Table comment 1: For many disorders, there exist functional tests that are considered gold or reference standard for diagnostic purposes or for use as trial endpoints, e.g., visual fields in glaucoma, muscle strength in sarcopenia, dental examination of pocket depth for periodontitis, and glomerular filtration rate for kidney dysfunction. Here we only list methods based on* in vivo* imaging techniques that enable direct quantification of the underlying pathological process*

*Table comment 2: For many age-related disorders, there exist imaging protocols that target specific proteins involved in unique pathophysiology of diseases, such as tau or amyloid imaging for certain dementia disorders. Here we only propose imaging modalities and techniques that allow for assessment of multiple and divergent pathological process in tandem*

### Cardiovascular

#### Atherosclerosis (Cardiac and cerebral infarction, angina pectoris, peripheral artery disease)

Atherosclerosis is the leading cause of cardiovascular disease, which in turn is the most common cause of death in the Western world [14]. The atherosclerotic process begins in childhood and progresses throughout the lifespan. Manifestation of atherosclerotic-induced cardiovascular disease rarely occurs before middle age and increases in frequency with progressing age [15].

Atherosclerosis is caused by the accumulation of cholesterol in the arterial walls, leading to inflammation and development of plaques. Rupture of a plaque may cause thrombosis, total vessel occlusion, stenoses, and embolisms. In addition, the accumulation of plaques leads to a thickening of the vessel wall which restricts blood flow, and can cause diseases such as angina pectoris and peripheral artery disease. Conversely, occurrences of most cardiovascular diseases are rare in the absence of atherosclerosis [16].

There exist several approaches to assess atherosclerosis in vivo using imaging. The PET radioligand [^18^F]FDG can be used to image the inflammatory process in vessel plaques that is integral to plaque formation. This technique has been used as an outcome in clinical trials assessing interventions aimed at treating atherosclerosis [17, 18]. Imaging with [^18^F]NaF PET mirrors the calcification which occurs at a relatively late stage of the atherosclerotic process [19]. Furthermore, using an MRI pulse wave velocity protocol, the elasticity in large arteries can be assessed [20]. Pulse wave velocity has been used as endpoint in clinical trials of putative atherosclerosis treatment [21].

#### Cardiac hypertrophy/fibrosis (heart failure)

With progressing age, the morphology and function of the heart changes; the cardiac myocytes decrease in number but increase in size, while the collagen within the myocardium increases in amount and changes its physical properties (i.e., fibrosis) [22]. These changes thicken the myocardial wall (i.e., hypertrophy) and cause a general reduction in tissue elasticity, resulting in a slower diastolic filling pattern and an increased atrial contraction [23]. This decline in diastolic function and loss of elasticity contributes to the overall age-related reduction in physical capacity and can lead to manifest heart failure [22]. The variant most associated with these changes is denoted “heart failure with preserved ejection fraction” (HFpEF) [24].

Imaging with echocardiogram or MRI (for improved precision) enables assessment of age-related structural and functional changes in the heart. Structural assessments include measurements of atrial and ventricular wall thickness and volume. Functional assessments include flow parameters that measure diastolic function (E/e’ and lateral e’) and measurements of myocardial contractility (strain) [25]. Such measures have been used as endpoints in drug trials evaluating HFpEF treatments [26].

### Musculoskeletal

#### Cartilage degeneration (Osteoarthritis)

With increasing age, the articular cartilage in the large joints degenerates. For example, the annual cartilage volume reduction in the knee joint is around 3% among healthy, middle-aged individuals [27]. This corresponds to a steep age-related increase in the prevalence of osteoarthritis; by the age of 80 around 50% of all individuals meet the criteria for knee osteoarthritis [28]. While the pathophysiology of the disorder is multifactorial, the central component is loss of articular cartilage.

Using MRI, the cartilage volume in joints can be measured. Such an outcome has been used as the primary endpoint in clinical trials assessing putative disease modifying interventions in osteoarthritis [29, 30]. Although less established, it is also possible to use MRI to assess other age-related morphological changes in the knee joint [31] relevant for the development of the disorder, such as MRI 3D bone area and bone shape [32, 33].

#### Loss of bone mass (Osteoporosis)

Osteoporosis is a systemic skeletal disease where loss of bone tissue exceeds the formation of new bone. This process leads to a fragile skeletal structure which increases the risk of fractures [34]. With progressing age, trabecular bone is lost from all parts of the skeleton. Women show an earlier disease onset as well as more rapid bone loss than men [35]. The bone mineral density is a well-established proxy for bone loss and is a strong predictor for the risk of future fractures [34]. Bone mineral density is also an FDA-approved surrogate endpoint for evaluating osteoporosis treatments [36].

The reference imaging protocol for assessing bone mineral density is DXA [37]. The use of high-resolution qCT has, however, been suggested to provide better diagnostic and prognostic information with regard to bone loss [38, 39]. In addition, by using [^18^F]NaF PET, it has been suggested that even higher sensitivity can be achieved [40, 41].

#### Loss of muscular mass (Sarcopenia)

Sarcopenia is a progressive and generalized skeletal muscle disorder, defined by the loss of muscle mass, muscle weakening and poor physical performance [42]. With progressing age, an accelerated reduction of muscle mass occurs [43, 44]. The condition is associated with an increased risk of falls, functional decline, and frailty [45]. In the early stages of the disorder, loss of muscle mass is the only established diagnostic criterion [46, 47].

DXA allows for the estimation of muscle mass in the whole body [48]. While DXA is the most frequently used technique to measure changes in muscle quantity, CT and MRI have been proposed to offer several advantages in the early detection, diagnostics, and monitoring of sarcopenia [47, 49]. These include the possibility to assess muscle quality, such as adipose tissue infiltration into muscular structures and intramyocellular lipid accumulation (measured with ^1^H-MRS) [50, 51], in addition to more precise muscle volume estimation [47, 52]. Imaging outcomes from CT and MRI have been recommended as surrogate endpoints for phase II studies evaluating treatment of sarcopenia [50].

### Metabolism

#### Visceral and ectopic fat deposition (Metabolic syndrome)

Changes in body composition, such as increases in body fat mass and decreases in lean body mass, occur with progressing age [53]. These changes include visceral and ectopic fat depositions, which are the key components in developing metabolic syndrome [54]. Metabolic syndrome is a common condition, and its prevalence is correlated with age [55–57]. The condition is a major risk factor for type 2 diabetes and cardiovascular disease [58]. It has been demonstrated that the risk for metabolic syndrome decreases with lower body fat mass and higher lean body mass [59].

Visceral and ectopic fat content can be imaged using DXA, CT, or MRI, using similar protocols to those described in the sarcopenia section above. DXA has been used in trials evaluating treatments for metabolic syndrome [60–62], and body composition MRI has been used in trials evaluating weight loss interventions [63–65].

#### Insulin resistance (type 2 diabetes)

Insulin resistance is defined as a systemic failure of cells to properly respond to insulin. In the periphery, insulin promotes glucose transport from blood into the cells where the molecule is metabolized into ATP. The condition is caused by a downregulation of the insulin receptor and a reduced function of the remaining receptors. There exists a multitude of downstream effects of this condition, and ultimately, as the pathology progresses, the pancreatic beta-cells become unable to produce sufficient amounts of insulin which marks the transition into type 2 diabetes. Several studies report associations between insulin resistance and aging [66–69].

Changes in glucose metabolism following the development of insulin resistance can be assessed using [^18^F]FDG PET. Insulin resistance may develop at different rates or intensities across distinct tissues, leading to heterogeneity within the cardiometabolic profiles (e.g., in the liver, compromised insulin signaling leads to elevated or less-inhibited hepatic glucose production, while insulin resistance in skeletal muscle results in reduced efficiency of glucose uptake by myocytes) [70]. To assess tissue-specific glucose utilization in relation to insulin resistance, the state-of-the-art technique is to perform a whole-body PET scan while the participant is subjected to a hyperinsulinemic euglycemic clamp [71].

#### Fatty liver and liver fibrosis (MASH/MAFLD, hepatocellular carcinoma, cirrhosis)

Metabolic dysfunction-associated fatty liver disease (MAFLD) covers a spectrum of disorders caused by abnormal accumulation of hepatic fat. Metabolic associated steatohepatitis (MASH) refers to a state in which the immune system triggers an inflammatory response to the fat deposits and marks a critical progression of the disorder. A common consequence of immune activation in the fatty liver is the generation of fibrotic tissue (fibrosis). Cirrhosis occurs when an excessive amount of fibrotic tissue has formed in the liver, causing impaired liver function. This has been demonstrated to increase the risk of hepatocellular carcinoma [72]. MAFLD is identified as a common, and probably underdiagnosed [73], condition among the aging population [74].

The presence of fibrosis in the liver causes tissue stiffness, which can be assessed using magnetic resonance elastography [75]. Liver fibrosis can also be assessed using PET. There currently exist PET radioligands for imaging the fibroblast activation protein inhibitor (FAPI), and platelet-derived growth factor receptor beta (PDGFRβ). FAPI and PDGFRβ PET are used to estimate the degree of fibrotic load and fibrosis formation, respectively [76, 77].

### Neurological

#### Neurodegeneration (Dementia disorders)

Neurodegeneration refers to a progressive loss of brain cells. Post-mortem studies have revealed a multitude of age-related effects in the human brain, most notably loss of gray matter tissue [78]. Neurodegeneration occurs as part of normal aging, although the rate varies across individuals and lifespan [79]. A common class of neurodegenerative disease is dementia disorders, such as Alzheimer’s Disease, which typically show a late-life onset. In these disorders, the degenerative process occurs at an accelerated rate and is associated with an accelerated cognitive decline.

MR imaging of the brain using a T1-weighted protocol can be used to estimate several outcome measures that reflect neurodegeneration, including gray matter volume and thickness, as well as ventricle enlargement. Further, arterial spin labelling MRI protocols can be used to assess the decline in cerebral perfusion following age-related vascular changes. In addition to the changes detectable using MRI, [^18^F]FDG PET can be used to assess neurodegeneration. A reduced [^18^F]FDG uptake indicates diminished glucose metabolism, which has been demonstrated to occur among elderly individuals and patients with neurodegenerative disorders [80]. Brain [^18^F]FDG uptake has been used as a surrogate endpoint in clinical trials assessing treatments for e.g., dementia disorders [81–84].

#### Loss of retinal ganglion cells and drusen accumulation (glaucoma, macular degeneration)

Several structural changes occur in aging eyes. In the retina, an overall thinning with age is well described. Specifically, the loss of ganglion cells causes the retinal nerve fiber layer to become progressively thinner with age [85]. This process is central in glaucoma, a common age-related ophthalmological disease, and causes visual field impairment [86]. In addition to the loss of ganglion cells, the aging eye also exhibits accumulation of extracellular proteins and lipid deposits between the retinal pigment epithelium and the Bruch’s membrane. These damaging formations, known as *drusen*, can be observed in most people over the age of 60. The presence of drusen is a prerequisite for developing age-related macular degeneration, which is the most common cause for age-related blindness [87].

Optical coherence tomography (OCT) allows for non-invasive in vivo imaging of sub-surface retinal structures with micrometer resolution. OCT measurements of the retinal nerve fiber layer and drusen deposits are used in the clinic for diagnosis and monitoring of disease progression [88, 89]. It has also been used as endpoint in clinical trials evaluating treatments for ophthalmological disorders [90–92].

### Dental

#### Periodontal inflammation and loss of tooth-bearing bone (Periodontitis)

Inflammation in the tissues surrounding the teeth increases with age and can lead to a progressive loss of tooth-supporting structures, including alveolar (periodontal) bone [93]. In severe cases, the condition is referred to as periodontitis, which is the most common cause of age-related loss of teeth. A majority of individuals over the age of 60 fulfill the criteria for periodontitis [94]. Loss of periodontal tissue manifests clinically as a periodontal “pocket”. The probing depth of the pocket is approved by the FDA as a surrogate endpoint for clinical trials assessing interventions against periodontitis.

Several imaging techniques can be applied for assessment of periodontal inflammation and bone loss. Using X-ray radiography, such as CT, it is possible to directly assess the loss in tooth bearing bone, that manifests in periodontal pockets. Furthermore, the occurrence and size of inflammation-induced edema can be quantified using MR imaging with a T2 STIR protocol [95]. [^18^F]FDG PET has also been used to assess ongoing periodontal inflammation [96].

### Urological

#### Hyperplasia of prostatic tissue (Benign prostatic hyperplasia)

The volume of the prostate gland increases with progressing age. Longitudinal studies report an average annual growth rate of around 2% in older men [97, 98]. This enlargement of the prostate gland can cause partial or complete obstruction of the urethra, leading to chronic lower urinary tract symptoms (LUTS), but also more acute conditions like urinary tract infections, urinary retention, and renal failure [99]. Change in prostate volume has been used as an outcome in clinical trials assessing interventions targeting benign prostatic hyperplasia [100].

The volume of the prostate gland can be assessed using MRI. This technique is commonly used both in clinical and research practices [101].

#### Loss of kidney function (Chronic kidney disease/Kidney failure)

The kidney’s ability to filter toxins and waste products from the blood markedly declines with progressing age [102, 103]. The functional decline starts around the third or fourth decade, and glomerular filtration rate measurements suggest around 5% reduction per decade thereafter [103, 104]. Severely decreased kidney function is associated with mortality [105–107].

MR imaging enables the assessment of the functional and structural state of kidneys in vivo, providing information beyond what can be obtained from blood and urine analyses [108]. This includes estimates of renal artery blood flow and resistive index, renal perfusion and filtration fraction, parenchyma and cortex volume, fat content, water diffusivity, and local oxygenation and inflammation [109]. Changes in these outcomes have been used as endpoints in clinical trials evaluating treatment of kidney disease [110–115].

## Discussion

In a recent paper surveying longevity researchers, the lack of valid biomarkers for evaluating CGPI efficacy in humans was identified as the most pressing issue for the field [116]. In the current review, we have outlined a set of medical imaging techniques that allows for in vivo assessments of age-related pathological processes present prior to disease onset. These techniques are readily available at most modern hospitals and can be combined to create an imaging battery for effective screening of CGPIs by detecting changes in pathology accumulation.

**Fig. 2:**
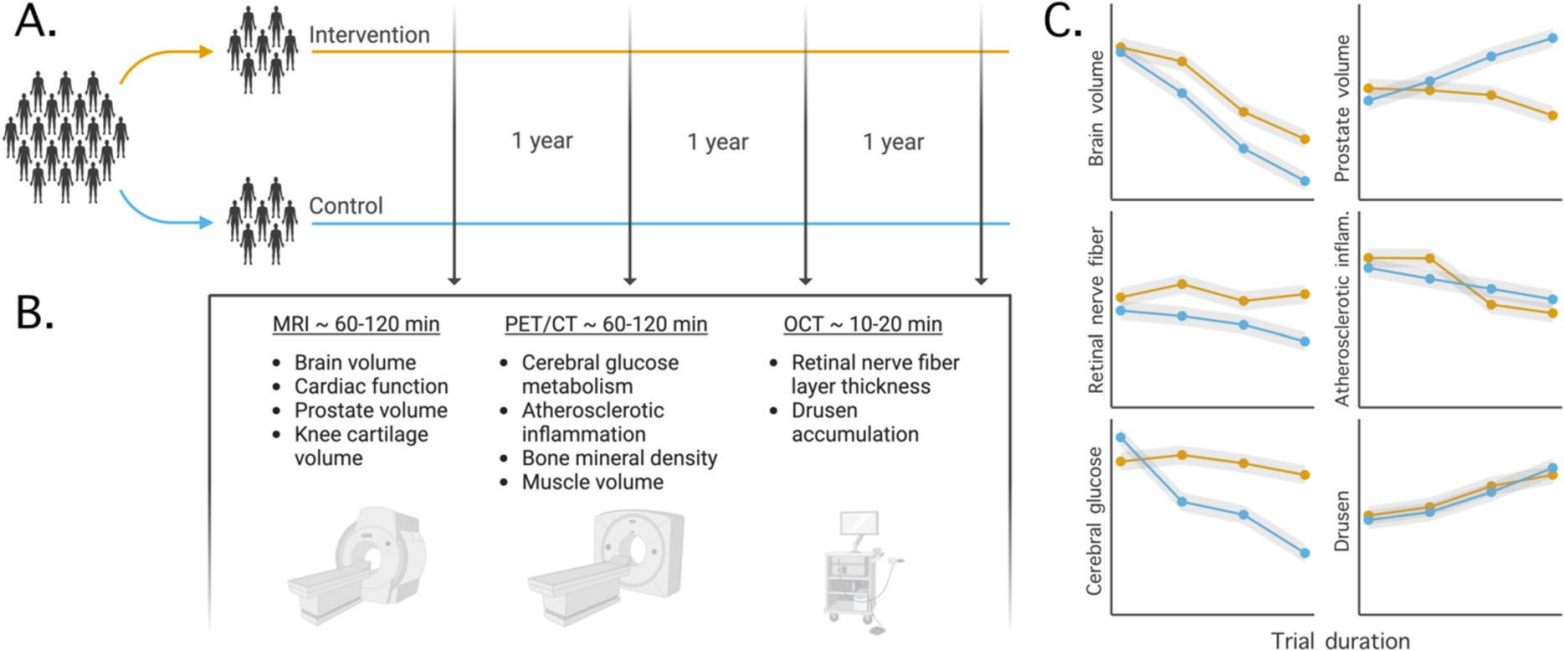
(**A**) An example design of a randomized controlled clinical trial with imaging outcomes. The number of imaging occasions can be varied to increase power (here, in vivo imaging is done at baseline, year 1, year 2 and at trial conclusion). (**B**) An example of a diverse imaging battery designed for detecting an effect of a geroprotective intervention. (**C**) Schematic of potential results from the trial. Here the presence of an effect in several age-related tissue pathologies would be interpreted as support that the treatment holds a general geroprotective effect. The largest effect is observed for the prostate gland, showing a decrease in volume (i.e., a reversal of pathology). This data could be used to consider benign prostatic hyperplasia as a suitable indication for regulatory approval

The use of medical in vivo imaging to aid drug development is not new. For decades, pharmaceutical companies have successfully included imaging as part of their development pipelines [117]. However, instead of focusing on a single tissue or indication, which is the usual practice in drug development, we propose to study multiple pathological processes in parallel. This builds on the geroscience hypothesis, which states that a geroprotective drug should have a beneficial effect on age-related pathologies in the whole body. For this reason, a battery including a suitable subset of protocols from Table 2 can provide a set of diverse outcomes that together reflect a general state of age-related pathology accumulation (see Fig. 1 and Fig. 2). The pathological processes described (such as atherosclerosis or neuronal loss) are prerequisites for the onset of age-related diseases (such as myocardial infarction or Alzheimer’s Disease). Assuming that a CGPI results in, for example, reduced plaque formation and increased elasticity in blood vessels, increased cartilage volume in the knee and reduced periodontal inflammation, this would suggest that the intervention possesses a broad geroprotective effect. Conversely, if no reversal or reduction of pathology accumulation can be seen in a well-powered controlled trial, the drug is unlikely to have a general beneficial effect on longevity and should not be advanced to subsequent phases.

In a recent review paper by the Biomarkers of Ageing Consortium, the efforts of identifying aging biomarkers suitable for drug development were summarized [4]. In this review, the emphasis was put on fluid biomarkers, such as proteomic assays or epigenetic clocks. Using a battery of in vivo imaging presents a distinctly different strategy. The imaging-based outcomes listed in Table 2 allow for *direct* assessment of detrimental age-related pathologies that precede morbidity and mortality. They thus come with an inherent mechanistic transparency and high biological plausibility. In the eyes of regulatory authorities, such as the FDA, these are key components for surrogate endpoints [13], and conceptually differentiate imaging based CGPI assessments from, for example, epigenetic clocks. Aging is however currently not an indication for which interventions can obtain approval by the FDA. Stakeholders in geroprotective drug development have therefore proposed to evaluate CGPIs for age-related disorders for which an established path to FDA approval already exists [118]. In addition to assessing the efficacy of a CGPI, a well-balanced battery may help to single out which approved indication is most suitable in this setting, by identifying the endpoints that show the strongest effect (see Fig. 2).

This imaging-based approach, however, is not without limitations. First, while an intervention that does not show an effect in any of the proposed outcomes (in a well-powered study) is unlikely to be an effective CGPI, the opposite is not obviously true. There are, for instance, important age-related processes that are difficult to assess using existing imaging techniques, such as the development of cancers. An intervention that shows a beneficial effect for all outcomes listed in Table 2 could still increase the incidence of e.g., cancer, and hence, mortality. Such an intervention is not a true GPI but would be classified as such using the proposed approach. Another possibility is that the proposed imaging battery could classify a compound as a successful GPI, although it has no influence on the fundamental biological aging process, but rather affects downstream processes. For example, a drug leading to a reduction in adipose tissue alone could lead to observable changes in insulin resistance, kidney function and cardiac function without affecting cellular aging. To minimize this risk, the protocols listed in Table 2 include outcomes quantifying a diverse set of pathologies, with a clear connection to aging. Second, for many of the outcomes listed in Table 2, there is a lack of data on the longitudinal test–retest reliability, especially in cohorts without manifest disease. This is a drawback, as without such information, it is difficult to calibrate sample sizes for sufficient power when designing a clinical trial. One critical task for future research is therefore to assess the test–retest reliability of the proposed protocols. This could also inform on suitable trial durations. Third, the proposed approach requires that at least some amount of age-related pathology is present in the body so that change can be reliably detected by the imaging techniques. Thus, the choice of suitable protocols for a screening battery will, to a certain extent, be limited by the age range of the study cohort. A final limitation is the use of imaging modalities involving ionizing radiation. The cumulative radiation exposure from repeated PET, low-dose CT, and qCT scans could potentially exceed low-risk thresholds defined by the International Commission on Radiological Protection. While such exposure can be ethically justified for addressing significant scientific questions, and while the inclusion of elderly participants partially mitigates concerns associated with radiation, it remains an important consideration in study design and participant safety. Furthermore, some pathological processes listed in Table 2 can be assessed using non-imaging procedures. For example, the reference standard to assess loss of tooth-bearing bone is a clinical examination by a dentist. If a study participant, however, is already being examined using CT, MRI and/or PET for other indications, the added cost to assess periodontal status with imaging is relatively small.

**Fig. 3:**
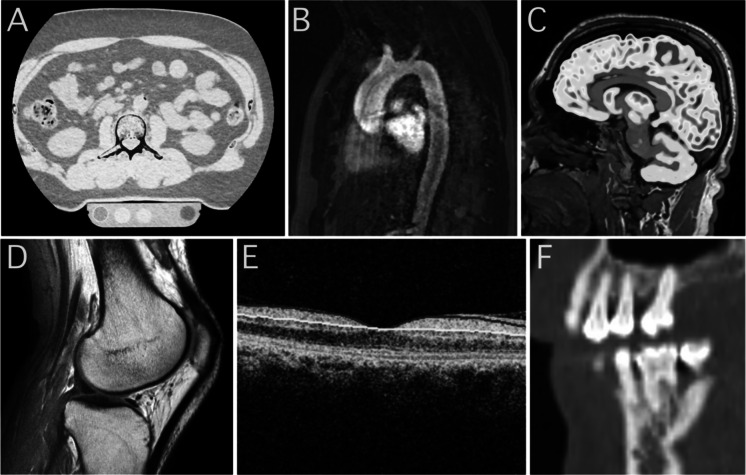
Example images from our ongoing clinical trial “Evaluating the effect of rapamycin treatment in Alzheimer’s disease and aging using in vivo imaging” [119] that includes a subset of protocols from Table 2. (**A**) Bone mineral density using computed tomography, axial image over the second lumbar vertebrae with the CT phantom used for absolute quantification visible in the lower part of the image. (**B**) Heart MRI for calculation of myocardial volume, diastolic function, and strain. (**C**) Brain MR with an overlaid [^18^F]FDG PET image for quantification of gray matter volume and cerebral glucose metabolism, respectively. (**D**) MR image of the knee for assessment of joint cartilage volume. (**E**) Optical coherence tomography, for measurement of retinal nerve fiber layer thickness and drusen accumulation, (**F**) CT image of tooth bearing bone status

The protocols listed in Table 2 primarily involve the use of CT, PET and MRI, and to a lesser extent, DXA and OCT. Acquisition and analysis of such imaging data are often associated with high costs, especially if study participants undergo multiple scanning sessions on different modalities. However, if the outcomes are sensitive and reliable enough, large sample sizes can likely be avoided while still providing evidence for the efficacy of an intervention. In addition, using modern integrated PET/CT or PET/MR systems, allowing for whole-body scanning with protocols of short acquisition time, it is possible to collect imaging data on multiple age-related processes in tandem. For instance, during a ~ 1 h long [^18^F]FDG PET/CT session, it is feasible to obtain measurements of glucose metabolism in the brain, periodontium, and large arteries, as well as density and structural measures of muscle, appendicular, axial, and tooth-bearing bone. Example images from a screening battery included in our ongoing phase IIa trial [119] of the CGPI rapamycin are shown in Fig. 3. Such multimodal and multi-organ data collection has also been performed in several longitudinal research initiatives with large sample sizes [120, 121].

Table 2 is not a comprehensive list of all the pathological processes that are relevant to include in a CGPI screening process; nor is it a complete list of all available imaging techniques to assess the included pathologies. For instance, imaging of misfolded proteins in the brain is today considered gold standard for diagnostics of dementia disorders such as Alzheimer’s disease [122]. Rather, we have selected a subset of processes and outcomes, which we consider to be suitable for a broad assessment age-related pathology over time, according to the specified criteria (see “Criteria for efficient screening of CGPIs” above). The aim of this paper has been to introduce the concept of multi-tissue and multi-modal imaging in order to translate promising preclinical CGPIs to humans, and not to provide a “final” list of protocols for future trials. The specific protocols to be selected and combined in a given study will depend on test–retest reliability assessments and longitudinal data on the rate of pathology accumulation in normally aging individuals.

## Conclusion

We propose a battery of medical imaging protocols that allow for in vivo assessments of age-related pathological processes prior to disease onset, which can be used for effective screening of CGPIs by detecting changes in pathology accumulation. This approach builds on the geroscience hypothesis, which states that a geroprotective drug should have a beneficial effect on age-related pathologies in the whole body. The proposed imaging battery consists of outcomes from modalities that are readily available at most modern hospitals and are already in use in drug development. As such, we submit that in vivo medical imaging offers an opportunity to evaluate the efficacy of geroprotective interventions today.
